# scPanel: a tool for automatic identification of sparse gene panels for generalizable patient classification using scRNA-seq datasets

**DOI:** 10.1093/bib/bbae482

**Published:** 2024-09-30

**Authors:** Yi Xie, Jianfei Yang, John F Ouyang, Enrico Petretto

**Affiliations:** Programme in Cardiovascular and Metabolic Disorders, Centre for Computational Biology, Duke-NUS Medical School, 8 College Road, Singapore 169857, Singapore; The School of Mechanical and Aerospace Engineering and the School of Electrical and Electronic Engineering, Nanyang Technological University, 50 Nanyang Ave, Singapore 639798, Singapore; Programme in Cardiovascular and Metabolic Disorders, Centre for Computational Biology, Duke-NUS Medical School, 8 College Road, Singapore 169857, Singapore; Programme in Cardiovascular and Metabolic Disorders, Centre for Computational Biology, Duke-NUS Medical School, 8 College Road, Singapore 169857, Singapore

**Keywords:** single-cell RNA-seq, machine learning, deep learning, marker panel, patient classification, clinical biomarkers

## Abstract

Single-cell RNA sequencing (scRNA-seq) technologies can generate transcriptomic profiles at a single-cell resolution in large patient cohorts, facilitating discovery of gene and cellular biomarkers for disease. Yet, when the number of biomarker genes is large, the translation to clinical applications is challenging due to prohibitive sequencing costs. Here, we introduce scPanel, a computational framework designed to bridge the gap between biomarker discovery and clinical application by identifying a sparse gene panel for patient classification from the cell population(s) most responsive to perturbations (e.g. diseases/drugs). scPanel incorporates a data-driven way to automatically determine a minimal number of informative biomarker genes. Patient-level classification is achieved by aggregating the prediction probabilities of cells associated with a patient using the area under the curve score. Application of scPanel to scleroderma, colorectal cancer, and COVID-19 datasets resulted in high patient classification accuracy using only a small number of genes (<20), automatically selected from the entire transcriptome. In the COVID-19 case study, we demonstrated cross-dataset generalizability in predicting disease state in an external patient cohort. scPanel outperforms other state-of-the-art gene selection methods for patient classification and can be used to identify parsimonious sets of reliable biomarker candidates for clinical translation.

## Introduction

Single-cell RNA sequencing (scRNA-seq) is a powerful tool for exploring cellular heterogeneity in complex biosamples like peripheral blood mononuclear cells (PBMCs) [1]. With advancements in scRNA-seq and sample multiplexing technologies, such as SPLiT-seq, profiling large patient cohorts has become feasible [2, 3]. These technologies offer accurate and scalable profiling of hundreds of samples in a single experiment [4]. These population-level single-cell studies profiling the entire transcriptome present a unique opportunity for automatic classification of patients' disease states based on gene expression patterns. However, the high cost associated with sequencing the entire transcriptome limits broader clinical applications, necessitating the biomarker identification in a specific and easily accessible cell type. The goal of our biomarker discovery is to identify a minimal number of genes that can discriminate the group (label) of the patients, such as disease subtypes or treatment response, so that only a few genes need to be assayed by low-cost quantitative real-time polymerase chain reaction (qRT-PCR) or targeted scRNA-seq for incoming patients in clinics. There are three critical tasks that can facilitate clinical translation of such biomarker discovery: (i) selecting a limited number of genes as clinical biomarkers (sparsity); (ii) constructing a model with good patient classification performance using these biomarkers (predictivity); (iii) validating the biomarkers in the external patient cohort (robustness).

Machine learning and feature selection has been widely used in scRNA-seq analysis, particularly for cell-type annotation [5–7]. However, there exist no tools that can concurrently classify patient groups from single-cell data and identify sparse gene panels as candidate biomarkers. To achieve patient-level classification from single-cell data, several machine-learning approaches have been developed. CloudPred predicts patient outcomes by modeling the abundance of cell subpopulations using a Gaussian mixture model [8]. ProtoCell4P uses prototype-based neural networks to classify patients based on gene expression values [9]. However, both methods did not validate their classifiers in a separate patient cohort. Additionally, given that both methods use all genes for patient classification, they fall short in providing a sparse biomarker panel, which is important to reduce the cost of the assay for real clinical applications. To improve sparsity in the biomarker gene selection from scRNA-seq data, several approaches have been developed including ActiveSVM and COMET. ActiveSVM is a machine learning–based active forward selection approach where the misclassified cells from the previous iteration are used to select the next most important gene for inclusion into the gene panel [10]. COMET is a statistical method employing an XL-minimal hypergeometric test to exhaustively search for gene combinations distinguishing specific cell populations [11]. While both approaches improve on sparsity, no data were provided to validate the predictivity of these gene panels in classifying unseen patient samples.

To address these challenges, we developed the scPanel tool, a computational framework to identify a small set of genes with high predictive power (sparsity and predictivity) for patient-level classification using scRNA-seq data. To account for the cell type differences in contribution to patient classification, scPanel first selects cell type(s) responsive to perturbations by quantifying the separation between two patient groups in each cell type. Then, a wrapper-based feature selection method is used to automatically identify a sparse gene panel with the minimal number of genes for the selected cell type. The performance of the sparse gene panel is then evaluated by classifying a set of test patient samples, either from the same cohort or a new cohort (robustness). Patient-level classification is achieved by aggregating the prediction probabilities of cells associated with a patient using the area under the curve (AUC) score, thereby accounting for the varying contributions of cells to patient classification. Beyond these improvements, the design of scPanel enables automatic determination of the number of selected genes and integration with methods like Seurat V3 canonical correlation analysis (CCA) to remove batch effects for cross-dataset prediction.

To demonstrate the utility of scPanel, we applied it to three scRNA-seq datasets: diffuse scleroderma (dSSc), colorectal cancer (CRC), and severe SARS-CoV-2 (severe COVID-19) [12–14]. Subsequently, we validated the ability of scPanel-derived biomarkers to generalize to new datasets by applying the severe COVID-19 classifiers to two separate unseen datasets [15, 16]. scPanel-derived biomarkers show consistent superior performance in patient classification when compared to classical feature selection methods and state-of-the-art (SOTA) gene selection methods (COMET, ActiveSVM) designed for scRNA-seq data. The scPanel tool is available at https://github.com/carissaxie/scPanel as an open-source Python package.

## Materials and Methods

### Overview of scPanel

**Figure 1:**
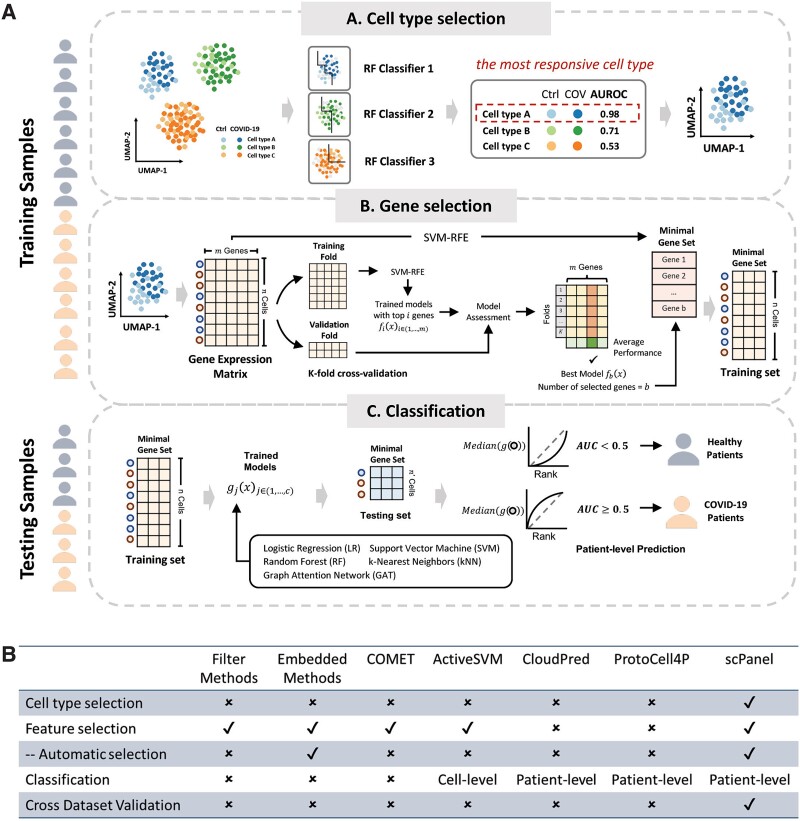
Workflow of the scPanel tool. (a) The scPanel tool comprises three main procedures. Cell-type selection: training samples (annotated with disease status labels, and cell types) are divided into internal training and validation datasets. An RF classifier is trained for each cell type and evaluated using the AUROC score to determine the impact of disease perturbation on each cell type. Gene selection: for the selected cell type(s), the minimal gene set is chosen using SVM-RFE based on k-fold cross-validation to ensure model parsimony. Classification model training and testing: the training and testing data are refined using the selected cell types and genes. Classification models are then trained, followed by computing the sample-level AUROC score, and a bootstrap *P*-value is calculated to predict each patient's disease status in the testing samples. (b) Summary table displaying the distinctive features and functionalities of scPanel compared to other approaches. Cross-dataset validation means successful patient classification achieved in a separate cohort using the predicted biomarker features.

We developed a machine learning based tool named scPanel to identify a sparse gene panel for patient-level classification of perturbations such as disease severity and treatment responses from scRNA-seq data (Fig. 1a, see Supplementary methods for technical details). scPanel comprises three key steps: (i) identifying the most responsive cell type to perturbation; (ii) selecting a minimal number of informative genes from this cell type using a wrapper-based feature selection method [17]; (iii) training multiple machine learning models with the selected cell types and genes for consensus patient-level classification.

scPanel starts by identifying responsive cell types in the scRNA-seq dataset, as gene expression changes post-perturbation may not occur in all cell types. This cell type identification step builds upon the method Augur [18], which assumes that more responsive cell types are more separable than less responsive ones. A random forest (RF) classifier is trained to predict the perturbation status for each cell type, with the area under the receiver operating characteristic curve (AUROC) score used to quantify prediction performance. A higher AUC indicates a larger separation in a particular cell type, corresponding to greater response to perturbations. However, a significant limitation of Augur is that it assumes that cells from different samples are identical, leading to the training and testing of the classifier on cells from the same sample. This assumption can result in the learning of sample-specific patterns during training, thereby inflating the cell-type responsiveness score. scPanel improves upon this by splitting training and testing data by samples, ensuring that the assessment of response to perturbation is generalizable to all patients. The process is repeated with an equal number of cells sampled from each patient. Higher weights are assigned to the minority class to reduce biases.

In the second step, scPanel constructs sparse gene panels for the selected cell type(s) through support vector machine recursive feature elimination (SVM-RFE), a wrapper-based backward elimination feature selection method [17]. Briefly, all available genes are used initially for classification and the least informative genes are then removed iteratively to search for the best combination of genes. This procedure is performed in a k-fold cross-validation manner to ensure that the number of selected genes is robust across patients. To improve efficiency, gene elimination has been implemented to reduce by percentage (default 3%) at each iteration. Inspired by the findPC tool [19], the minimal number of genes is automatically determined using the perpendicular line method, which corresponds to the gene count just before a rapid decline in classification accuracy (see Supplementary methods).

To evaluate the effectiveness of the predicted biomarkers, selected cell types and genes are used to train cell-level classifiers with five different machine learning or deep learning algorithms, logistic regression (LR), SVM, RF, k-nearest neighbors (kNN) and graph attention network (GAT) to account for different complexity within scRNA-seq datasets [20–24]. The median of the consensus prediction probabilities from the five classifiers is used for cell-level prediction. Patient-level classification is determined by aggregating cell prediction probabilities using the AUC score, with a corresponding *P*-value to quantify significance (see Supplementary methods). In scPanel, all classifiers are weighted by the number of cells in each patient and the number of cells in each class to avoid bias in dataset compositional differences.

Overall, the combination of four properties: (i) identifying the most responsive cell type(s), (ii) automatically selecting a minimal number of genes as biomarkers, (iii) constructing a patient-level classification model with high predictive performance using these biomarkers, and (iv) validating the performance of the biomarkers in the external cohort, makes scPanel an efficient and user friendly end-to-end tool to achieve biomarker discovery and patient classification in scRNA-seq data (Fig. 1b).

## Results

### Sparse gene panel identification and patient classification with scPanel

We applied scPanel on five scRNA-seq datasets: a dSSc skin dataset (gur2022ssc), a CRC dataset (joanito2022crc), two severe COVID-19 PBMC datasets (wilk2020covid, su2020covid), and an infection PBMC datasets (lee2020cov_flu; Supplementary Table 1). We tested scPanel in two settings: within-dataset prediction and cross-dataset prediction. In the within-dataset prediction task, gur2022ssc, joanito2022crc, and wilk2020covid datasets were split into a training set (80% of patients) and a testing set (20% of patients) for model training and evaluation, respectively. In cross-dataset prediction, we demonstrated the generalizability of scPanel to predict datasets generated by different laboratories by testing the performance of a model trained from the wilk2020covid dataset to predict patients in the su2020covid dataset. The specificity of this severe COVID-19 classifier was then assessed by making predictions on COVID-19 and influenza patients in the lee2020cov_flu dataset.

Each dataset was first preprocessed to remove low-quality cells and genes. Each cell's gene expression is normalized by the total Unique Molecular Identifier (UMI) counts using standard pipelines (see Supplementary methods). Cell types with at least 20 cells detected in at least 3 patients in each class are retained. For each analysis, patients in training and testing sets are not overlapped to ensure that there is no information leakage during cell-type selection, gene selection and classification. AUC score is calculated for each patient in the testing set to evaluate the classification performance along with the patient-level accuracy, specificity, precision, sensitivity, and F1 score. Details on the parameters used for each model are provided in Supplementary Table 2.

### Sparse gene panel for classifying scleroderma patients

**Figure 2:**
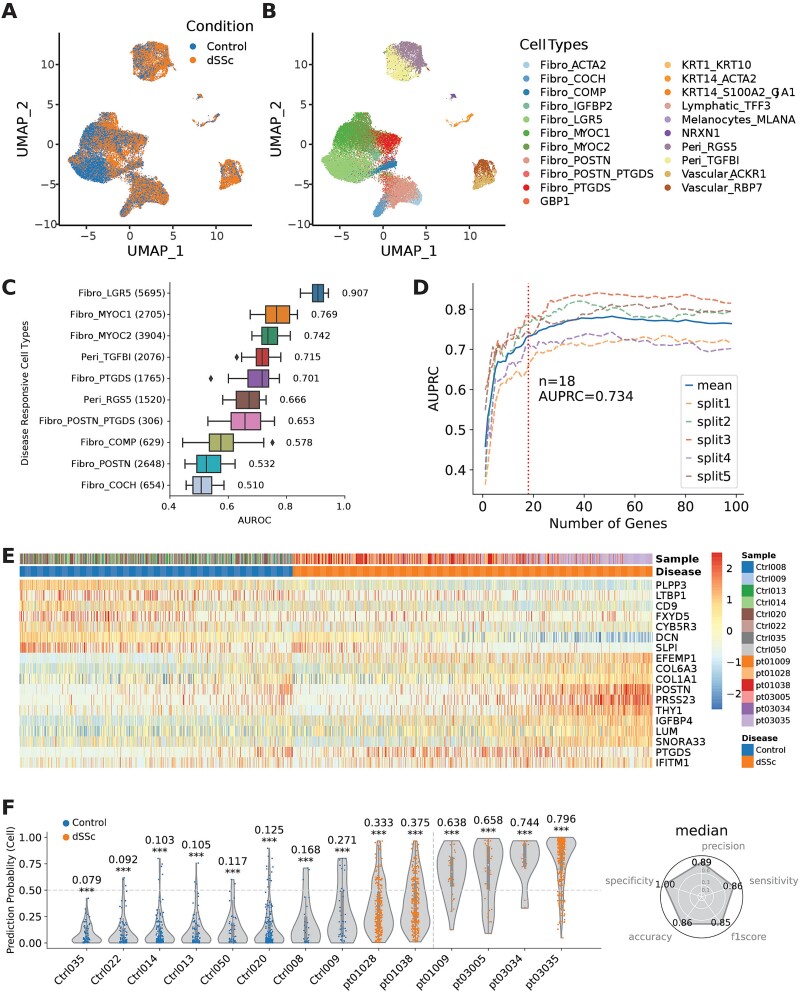
Using scPanel to classify dSSc patients. (a) UMAP of input dataset colored by conditions. (b) UMAP of input dataset colored by cell types. (c) Cell types ranked by disease responsive score (AUROC). (d) Parsimony plot of number of variables (genes) retained and average predictive performance of corresponding model over 5-folds, calculated for Fibro_LGR5 cell type. Dashed line shows the number of genes (*n* = 18) for achieving the parsimonious model. (e) Heatmap of gene expression of selected genes in the test set. Values are z-score of normalized gene expression truncated at ±2.5. (f) Violin plot of patient-level prediction result. Violins are colored by the label of patients. Y-axis represents cell-level prediction probability summarized by taking the median of five classifiers’ prediction probabilities. Value on top of each violin represents sample-level AUC score with bootstrap *P*-value (^*^P < .05; ^*^^*^P < .01; ^*^^*^^*^P < .001). The vertical line indicates the patient-level prediction threshold (AUC = 0.5). Samples at the right side of this line are predicted as dSSc patients. Accuracy, precision, sensitivity, and recall are computed to evaluate the overall patient classification performance.

To demonstrate scPanel's scalability to large scRNA-seq datasets with hundreds of patients, we applied the method to a dSSc skin dataset containing 35,417 cells from 91 donors (41 controls, 50 SSc patients; Fig. 2a, Supplementary Fig. 1a). Cell-type annotations from the original publication, 21 cell types in total, were input to scPanel (Fig. 2b). Different cell types exhibit varying degrees of response to disease perturbation, with *LGR5*-expressing fibroblasts (Fibro_LGR5) showing the strongest response with an AUC score of 0.907 (Fig. 2c). The analysis from scPanel is consistent with the finding in the original publication that the most substantial transcriptional changes in the fibroblast lineage were observed in the Fibro_LGR5 cell type from SSc patients [12].

We performed gene selection using the Fibro_LGR5 cell type with cells labeled by disease status. Eighteen genes were identified with 5-fold cross-validation, providing enough discriminative power to distinguish dSSc patients (Fig. 2d and e). All five classifiers trained with selected genes and the Fibro_LGR5 cells consistently showed good performance (Supplementary Fig. 1d). All patients are accurately classified except for pt01028 and pt01038 with AUC scores of 0.333 and 0.375, respectively. Overall, the patient-level classification accuracy is 0.86 (12 out of 14 patients) in the testing set (Fig. 2f, Supplementary Fig. 1c).

scPanel identified known and potentially new markers of dSSc within the LGR5-expressing fibroblasts (Fig. 2e, Supplementary Fig. 1b). The *POSTN* gene has been reported to contribute to pathogenesis of scleroderma via *PI3K/Akt*-dependent mechanism [25]. The *PLPP3* gene is associated with platelet aggregation in atherosclerosis, a complication common to many SSc patients [26, 27]. *PTGDS*, a gene important in prostaglandins synthesis, is predictive of poor survival in SSc at protein level [28]. scPanel also identifies multiple genes associated with excessive production and deposition of extracellular matrix (ECM) components (e.g. *COL6A3*, *COL1A1*, *LTBP1*, *EFEMP1*, *LUM*, *DCN*) in parallel with the uncontrolled tissue proteases activity (upregulation of the *PRSS23* gene and the downregulation of the *SLPI* gene). Excessive deposition of collagen and ECM proteins as well as the dysregulation of ECM components are known hallmarks of SSc, leading to skin and internal organ fibrosis [29]. The results show that scPanel can identify genes that are involved in SSc pathogenesis and define a sparse gene panel for accurate SSc patient classification.

### Sparse gene panel for classifying colorectal cancer patients

**Figure 3:**
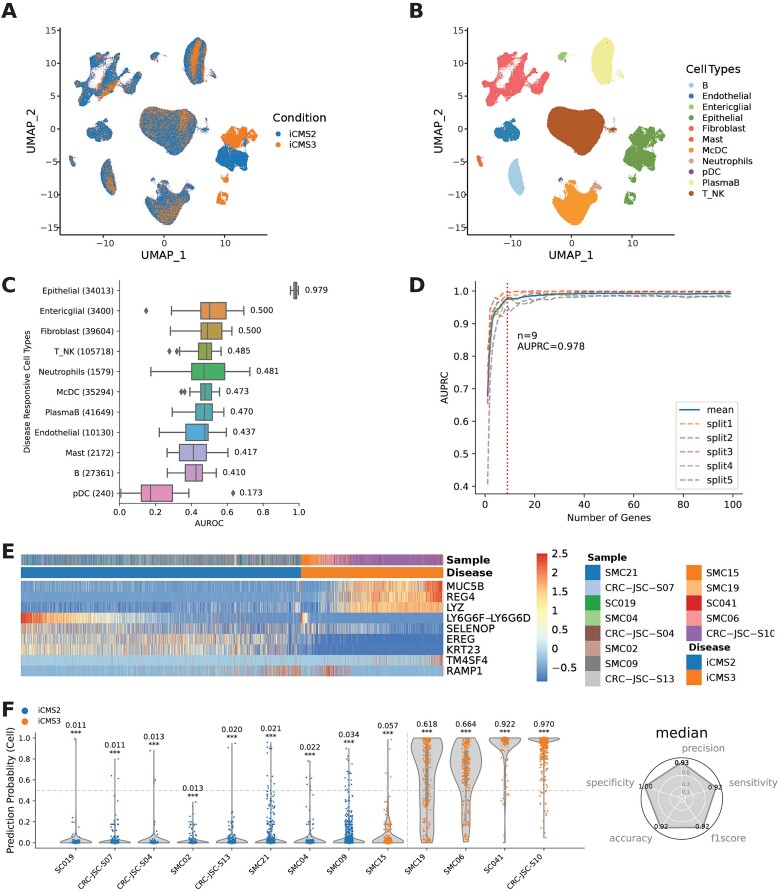
Using scPanel to classify iCMS2 and iCMS3 patients in CRC. (a) UMAP of input dataset colored by conditions. (b) UMAP of input dataset colored by cell types. (c) Cell types ranked by disease responsive score (AUROC). (d) Parsimony plot of number of variables (genes) retained and average predictive performance of corresponding model over 5-folds, calculated for epithelial cells. Dashed line shows the number of genes (*n* = 9) for achieving the parsimonious model. (e) Heatmap of gene expression of selected genes in the test set. Values are z-score of normalized gene expression truncated at ±2.5. (f) Violin plot of patient-level prediction result. Violins are colored by the label of patients. Y-axis represents cell-level prediction probability summarized by taking the median of five classifiers’ prediction probabilities. Value on top of each violin represents sample-level AUC score with bootstrap *P*-value (^*^P < .05; ^*^^*^P < .01; ^*^^*^^*^P < .001). The vertical line indicates the patient-level prediction threshold (AUC = 0.5). Patients at the right side of this line are predicted to be iCMS3. Accuracy, precision, sensitivity, and recall are computed to evaluate the overall patient classification performance (iCMS: intrinsic consensus molecular subtype).

To determine whether scPanel can classify patients with very heterogeneous diseases such as cancer, we applied the method to a CRC dataset comprising 365,913 cells from 7 distinct anatomical regions of the colon from 38 iCMS2 and 25 iCMS3 patients [13] (Fig. 3a, Supplementary Fig. 2a). Specifically, the authors identified two patient subtypes, iCMS2 and iCMS3 with different prognosis, and here we applied scPanel to identify a sparse gene panel distinguish these patient subtypes. Cell-type annotations (*n* = 11 cell types) from the original study were used as input (Fig. 3b). Most cell types did not show substantial gene expression changes between the two patient subtypes except for epithelial cells which have the largest response with AUC = 0.979 (Fig. 3c). This finding from scPanel is consistent with that of the original publication where the authors found two distinct malignant epithelial subtypes, corresponding to iCMS2 and iCMS3 subtypes. scPanel revealed that nine genes from epithelial cells are sufficient to distinguish the iCMS2 and iCMS3 subtypes (Fig. 3d). These genes showed significant gene expression differences (adjusted *P*-value <.05) between iCMS2 and iCMS3 subtype patients across both training and testing set (Supplementary Fig. 2b, Fig. 3e). Twelve out of 13 patients (92.3% accuracy) were predicted confidently with AUC scores significantly different from 0.5 (random guess; Supplementary Fig. 2c and Fig. 3f). All five classifiers consistently showed the same good performance (Supplementary Fig. 2d).

The nine genes identified by scPanel belong to the 715 DEGs previously reported [13]. This highlights how scPanel can identify a small set of genes from the whole set of DEGs, which is sufficient to classify the iCMS2 and iCMS3 subtypes. Moreover, many of these genes are associated with stemness, such as *MUC5B*, as well as tumor biology, including *REG4*, *LYZ*, *EREG*, *KRT23*, and *RAMP1*, which were also identified in a previous CRC single-cell study [30]. The identification of these genes in two CRC single-cell studies not only supports their biological relevance to tumor biology, but also emphasizes their potential as biomarkers in CRC subtypes iCMS2 and iCMS3.

### Sparse gene panel for classifying severe COVID-19 patients

**Figure 4:**
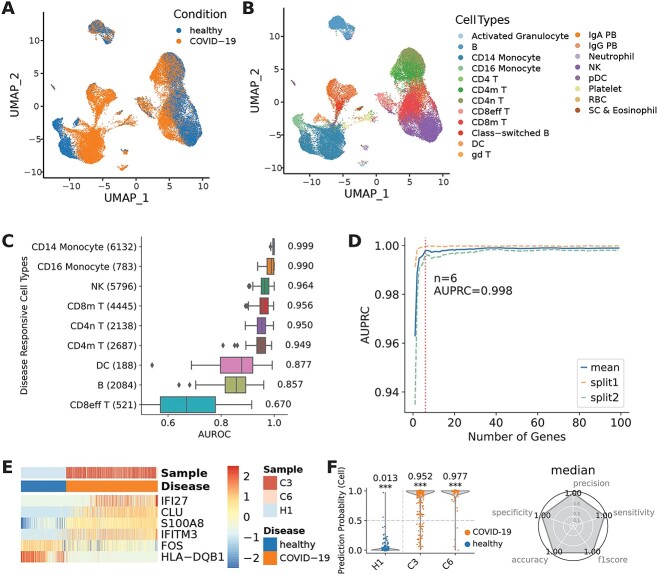
Using scPanel to classify severe SARS-CoV-2 (COVID-19) patients. (a) UMAP of input dataset colored by conditions. (b) UMAP of input dataset colored by cell types. (c) Cell types ranked by disease responsive score (AUROC). (d) Parsimony plot of number of variables (genes) retained and average predictive performance of corresponding model over 2-folds, calculated for CD14+ monocytes cell type. Dashed line shows the number of genes (*n* = 6) for achieving the parsimonious model. (e) Heatmap of gene expression of selected genes in the test set. Values are z-score of normalized gene expression truncated at ±2.5. (f) Violin plot of patient-level prediction result. Violins are colored by the label of patients. Y-axis represents cell-level prediction probability summarized by taking the median of five classifiers’ prediction probabilities. Value on top of each violin represents sample-level AUC score with bootstrap *P*-value (^*^P < .05; ^*^^*^P < .01; ^*^^*^^*^P < .001). The vertical line indicates the patient-level prediction threshold (AUC = 0.5). Patients at the right side of this line are predicted to be severe COVID-19 patients. Accuracy, precision, sensitivity, and recall are computed to evaluate the overall patient classification performance.

To investigate the wider applicability of scPanel, we applied the method to a severe COVID-19 dataset with 39,196 PBMCs from 13 patients (6 controls, 7 COVID-19 patients) [14] (Fig. 4a, Supplementary Fig. 3a). Cell-type annotations (*n* = 20 cell types) from the original literature were used as input (Fig. 4b). Most of the cell types have substantial gene expression changes after severe COVID-19 perturbation. CD14+ monocytes showed the strongest immune response with an AUC score = 0.999 (Fig. 4c). scPanel showed that six genes from CD14+ monocytes were sufficient to provide a high accuracy to identify severe COVID-19 patients (Fig. 4d). These six genes (*IFI27*, *CLU*, *S100A8*, *IFITM3*, *FOS*, and *HLA-DQB1*) showed consistent gene expression patterns between training and testing set (Supplementary Fig. 3b, Fig. 4e). All patients were predicted confidently with AUC scores significantly different from 0.5 (random guess; Supplementary Fig. 3c). This resulted in 100% accuracy (3 out of 3 patients) with AUC scores 0.013 for H1, 0.952 for C3 and 0.977 for C6, respectively (Fig. 4f). All five classifiers consistently showed the same good performance (Supplementary Fig. 3d).

The genes identified by scPanel play important roles in the immune response and inflammatory processes, which are key determinants in the progression of COVID-19. For example, *S100A8* encodes an alarmin protein that mediates host pro-inflammatory responses during infection [31–33]. It has been shown that *S100A8* is responsible for the induction of an aberrant neutrophil subset in pathogenesis of COVID-19 through stimulating the *TLR4* signaling [34], which are thought to induce cytokine storm or excessive inflammation, as is observed in severe COVID-19 cases [35–37]. *IFITM3* belongs to the interferon-induced transmembrane (IFITM) protein family that is known to play a significant role in antiviral response by restricting virus entry into cells [38–45]. It has been recently reported that SARS-CoV-2 virus hijacks IFITM proteins for efficient infection, which explains the rapid spread of this virus [46].

### Generalizability of prediction of severe COVID-19 patients

**Figure 5:**
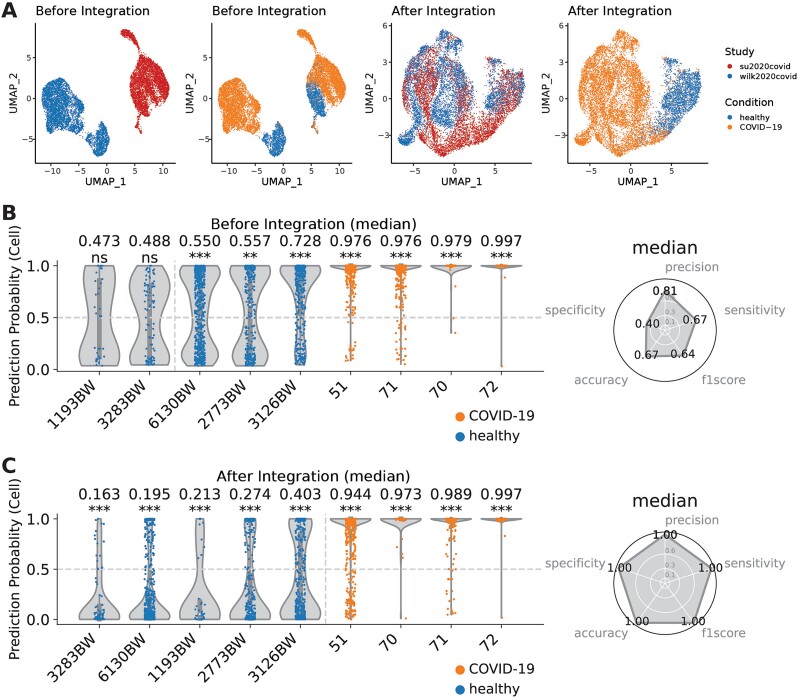
Using scPanel to predict cross-batch severe SARS-CoV-2 (COVID-19) patients. (a) UMAP of CD14+ monocytes from training data (wilk2020covid) and testing data (su2020covid) before integration and after integration, colored by study or condition. (b and c) Violin plot of patient-level prediction results in testing data (su2020covid) (b) without data integration and (c) after data integration. Violins are colored by the label of patients. Y-axis represents cell-level prediction probability summarized by taking the median of five classifiers’ prediction probabilities. Value on top of each violin represents sample-level AUC score with bootstrap *P*-value (^*^P < .05; ^*^^*^P < .01; ^*^^*^^*^P < .001). The vertical line indicates the patient-level prediction threshold (AUC = 0.5). Patients at the right side of this line are predicted to be severe COVID-19 patients. Accuracy, precision, sensitivity, and recall are computed to evaluate the overall patient classification performance.

To demonstrate the generalizability of scPanel across different datasets, we applied the severe COVID-19 classifiers to an independent su2020covid dataset (Supplementary Fig. 4a and b). Specifically, CD14+ monocytes were computationally isolated from the su2020covid dataset in concordance with the cell type used in the training data. We observed pronounced batch effects between the training set (wilk2020covid) and the testing set (su2020covid) with a clear separation of cells by their dataset-of-origin (Fig. 5a). Consequently, the classifier exhibited a suboptimal performance with an accuracy of 0.67, misclassifying all healthy patients (Fig. 5b).

We hypothesized that these batch effects need to be removed in order to achieve an accurate prediction. To address this, we applied the Seurat V3 CCA method to remove batch effects for better cross-dataset prediction performance [46]. After removing batch effects from the testing set, we observed a more integrated representation of CD14+ monocytes from both datasets, with a predominant clustering of cells by disease status rather than dataset-of-origin (Fig. 5a). This procedure substantially improved the cross-dataset prediction accuracy, which increased from 0.67 to 1.00 (Fig. 5c). Interestingly, the GAT classifier showed an inherent robustness to batch effects, consistently maintaining 100% accuracy, irrespective of batch removal integration (Supplementary Fig. 4c and d). Our findings show that scPanel, providing appropriate batch effect removal, can serve as a tool for patient-level classification in cross-dataset prediction settings.

### Specificity of prediction of severe COVID-19 patients

**Figure 6:**
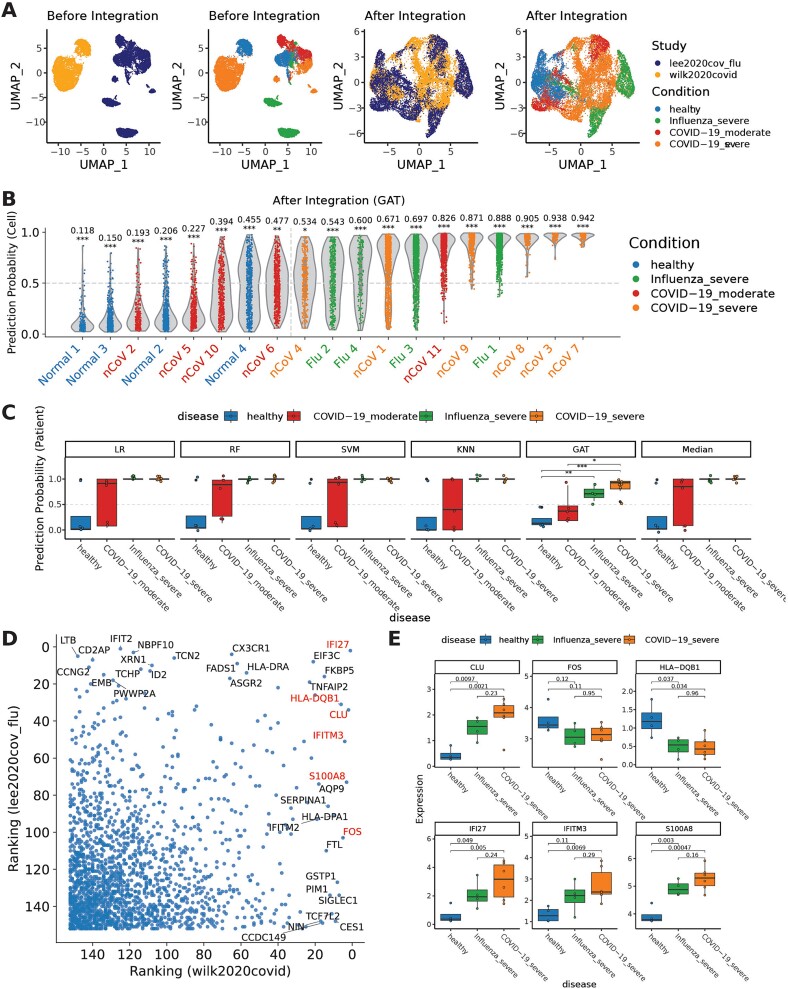
Specificity of severe SARS-CoV-2 (COVID-19) biomarkers derived from wilk2020covid dataset by scPanel. (a) UMAP of CD14+ monocytes from training data (wilk2020covid) and testing data (lee2020cov_flu) before integration and after integration. (b) Violin plot of patient-level prediction result of GAT classifier in testing data (lee2020cov_flu) after data integration. Violins are colored by the label of patients. Y-axis represents cell-level prediction probability. Value on top of each violin represents sample-level AUC score with bootstrap *P*-value (^*^P < .05; ^*^^*^P < .01; ^*^^*^^*^P < .001). Vertical line indicates the patient-level prediction threshold (AUC = 0.5). Patients at the right side of this line are predicted as severe COVID-19 patients. (c) Box plots showing patient-level prediction probability of severe COVID-19 summarized by taking median of cell-level prediction probabilities. (d) Scatter plot of gene importance ranking between severe COVID-19 (x-axis) and severe influenza (y-axis) compared to healthy controls. (e) Box plots showing expression levels of severe COVID-19 biomarkers in healthy controls, severe influenza, and severe COVID-19 patients. *P*-values indicate the significance of *t*-test performed between healthy and severe COVID-19 expression levels.

To assess the specificity of our severe COVID-19 classifiers (LR, RF, SVM, kNN, and GAT), we incorporated a PBMC dataset, referred to as lee2020cov_flu, from patients with various COVID-19 severities and including influenza cases (Supplementary Fig. 5a–c). If the severe COVID-19 gene set, along with the trained classifiers, specifically captures the unique expression pattern distinct from that of mild COVID-19 and influenza cases, the classifiers trained on healthy and severe COVID-19 data should yield prediction probabilities randomly distributed between 0 and 1 when predicting moderate COVID-19 and influenza cases. To obtain the prediction probabilities on lee2020cov_flu dataset, we first integrated it with our training dataset (wilk2020covid) to mitigate the batch effects (Fig. 6a). After integration, all five classifiers demonstrated a 100% accuracy in predicting healthy individuals and severe COVID-19 patients within the lee2020cov_flu dataset (Supplementary Fig. 5d and e). However, LR, RF, SVM, and kNN classifiers demonstrated a lack of specificity for severe COVID-19, incorrectly classifying all severe influenza cases as severe COVID-19 with 100% prediction probabilities (Supplementary Fig. 5e). In contrast, these analyses suggest that the GAT classifier can distinguish the minor differences in gene expression between severe COVID-19 and other conditions, assigning a median sample prediction probability of 0.65 to severe influenza cases and 0.45 to moderate COVID-19 cases (Fig. 6b and c).

To investigate the potential non-specificity of the gene set used in the severe COVID-19 classifier, we derived a severe influenza gene set from lee2020cov_flu by applying scPanel to healthy and severe influenza groups. We observed that certain genes in the severe COVID-19 gene set, such as *IFI27*, *HLA-DQB1*, and *CLU*, also hold significance in the context of influenza patient classification (Fig. 6d). Notably, *IFI27* has been previously described as a biomarker for various infectious diseases including COVID-19 and influenza [47, 48]. Furthermore, the expression levels of these severe COVID-19 genes exhibited no significant differences between severe influenza and COVID-19 groups (Fig. 6e).

### Benchmarking

**Figure 7:**
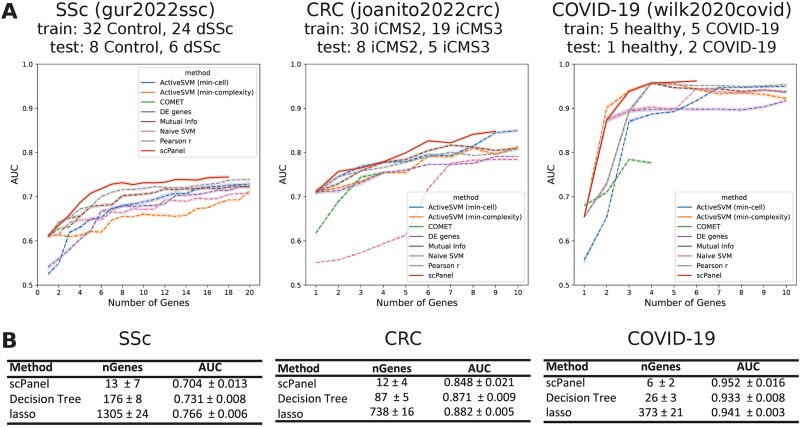
Benchmarking results of scPanel gene selection step. (a) The performance (AUC) of scPanel’ s feature selection is compared to other filter methods using top 1,2, …, 20 genes ranked in each method. Gene lists from filter method are evaluated 20 times by randomly sampling 50% of the cells from the same training dataset to generate 95% confidence intervals for AUC score. (b) the performance (AUC) of scPanel’ s feature selection compared to other embedded methods using genes automatically selected by each method. The evaluation is repeated 20 times by randomly sampling 50% of the cells from the same training dataset, mean ± SD is displayed. This was done to assess both the number of genes automatically selected and the corresponding performance in patient classification. SSc: scleroderma (gur2022ssc dataset), CRC: colorectal cancer (joanito2022crc dataset), COVID-19: severe SARS-CoV-2 (wilk2020covid).

For the feature selection function of scPanel, we compared the performance of using scPanel-identified genes as biomarkers for classifying patients, against genes identified by other feature selection methods, including filter-based (Pearson correlation, mutual information, differentially expressed genes), embedded (lasso regression, decision tree) and hybrid (naïve-SVM) approaches. For filter methods, each method generated a ranked list of genes, and we assessed classification accuracy by incrementally including the top-ranked genes from each list. Our analyses show that scPanel consistently provides better predictive performance in all datasets as the number of genes added to the classifier increases (Fig. 7a). For embedded methods, which automatically select gene panels during model training, scPanel selected 4–13 times fewer genes compared to decision tree and 10–100 times fewer genes than lasso while maintaining a similar predictive performance (Fig. 7b). To complete our benchmark, we performed a comparative analysis of computational cost (runtime) of scPanel and the other SOTA gene selection methods, COMET and ActiveSVM, across the three datasets (Supplementary Fig. 6). This analysis reveals that scPanel exhibits superior scalability compared to COMET but falls short of the scalability achieved by ActiveSVM (min_complexity), a method specifically designed for scalable gene selection through active learning [10].

### Stability analysis

**Figure 8:**
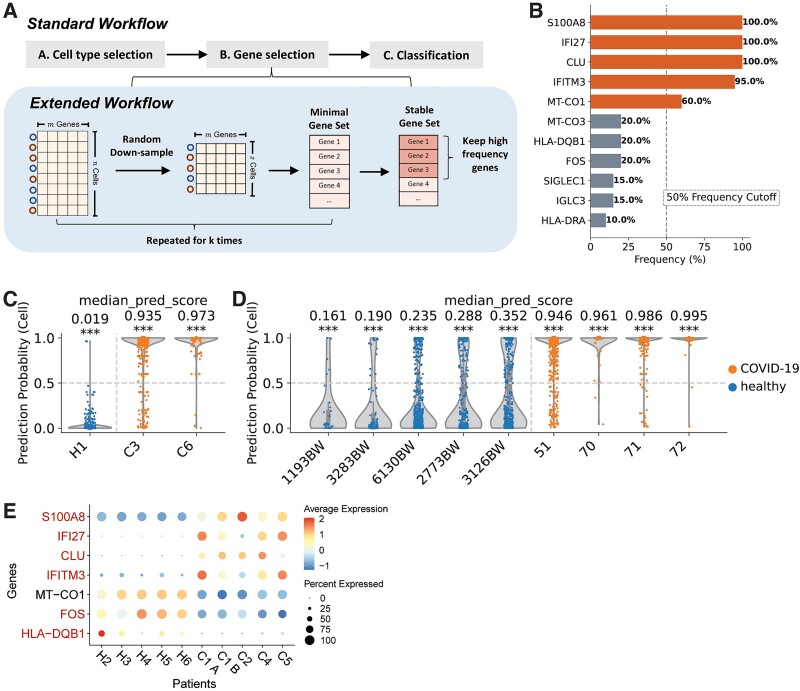
Implementation of stable gene selection in scPanel. (a) Stable gene selection workflow in scPanel. (b) Selection frequency of genes in severe COVID-19 training dataset (wilk2020covid) using stable gene selection workflow. Violin plot of patient-level prediction results in (c) within-batch data (wilk2020covid) and (d) cross-batch data (su2020covid). Violins are colored by the label of patients. Y-axis represents cell-level prediction probability summarized by taking the median of five classifiers’ prediction probabilities. Value on top of each violin represents sample-level AUC score with bootstrap *P*-value (^*^P < .05; ^*^^*^P < .01; ^*^^*^^*^P < .001). The vertical line indicates the patient-level prediction threshold (AUC = 0.5). Patients at the right side of this line are predicted to be severe COVID-19 patients. (e) Dot plot of average expression values of genes from standard gene selection (*S100A8*, *IFI27*, *CLU*, *IFITM3*, *FOS*, *HLA-DQB1*) and stable gene selection methods (all eight genes), applied to the same training data.

In the default scPanel gene selection procedure, selecting genes from a single training set might lead to overfitting, where the gene expressions describe patterns specific to the training set but do not generalize to other datasets. To improve the robustness of our gene selection methodology, we have implemented a stable gene selection mode in scPanel. This approach involves iterative downsampling of the training data into multiple subsets, followed by the application of the scPanel gene selection algorithm to each subset. This downsampling simulates the presence of multiple random cohorts, allowing for the identification of ‘stable genes’ that are consistently being selected across cohorts and thereby reducing the model variance. Genes that are consistently selected in over a fraction (e.g. 50%) of these subsets are retained, culminating in a final, more reliable gene panel (Fig. 8a).

Using this approach, genes *S100A8*, *IFI27*, *CLU*, *IFITM3*, and *MT-CO1* have been identified as the stable gene panel for the classification of severe COVID-19 patients, utilizing the same training dataset as previously employed (Fig. 8b). Classifiers developed using this stable gene panel (comprising five genes) demonstrated comparable performance to that trained with the standard procedure (comprising seven genes), achieving 100% accuracy in both within-dataset and cross-dataset predictions (Fig. 8c and d). Notably, genes like *FOS* and *HLA-DQB1*, which were selected in the standard procedure, were not chosen in the stable gene selection (Fig. 4e and Fig. 8b). Instead, *MT-CO1* was selected as part of the stable gene set. Expression levels of all selected genes were analyzed in each patient (Fig. 8e). *MT-CO1* exhibited consistent expression levels across patients in both the control and disease groups, whereas *FOS* and *HLA-DQB1* showed variable and inconsistent expressions, making them less favored by stable gene selection. This stable gene selection mode has been incorporated into our scPanel python package, allowing users to explore its impact on feature selection and prediction accuracy.

### Power analysis and computational requirements

To assess scPanel's performance with varying cell number and sample size, we conducted an empirical power analysis by downsampling the training data to different sizes. In the severe COVID-19 dataset (wilk2020covid), cell-level analysis consistently identified CD14+ monocytes as the most responsive cell type across all subsets (Supplementary Fig. 7a), with some variation in the identified genes (Supplementary Fig. 7b) but a smaller set of stably selected genes (Supplementary Fig. 7c). Despite this, predictions for all patients in the test set remained accurate across all subsets (lower bound AUC > 0.85), albeit with a marginal reduction in AUC scores for subsets with size smaller than 70% (Supplementary Fig. 7d). Further cell-level power analysis within the dSSc dataset (gur2022ssc) identified LGR5+ fibroblasts as the most responsive cell type when more than 13,141 cells (60% of the total) were used (Supplementary Fig. 8a). Above this number of cells, there were no significant differences in the number of selected genes with similar sets of genes being identified consistently (*P* = .18, Kruskal–Wallis test) and the patient-level predictions remain the same for LGR5+ fibroblasts (Supplementary Fig. 8b–d).

For sample-level power analysis, the dSSc dataset (gur2022ssc) was selected due to its large patient cohort. It was observed that the cell type responsiveness score (AUC) for LGR5+ fibroblasts began to plateau at 40% (33 samples) of the full sample size (Supplementary Fig. 9a). Correspondingly, the gene selection started to exhibit large variations when the sample sizes were smaller than 40% (Supplementary Fig. 9b and c). Despite the variability in gene selection, the top 5 most important genes (*DCN*, *PLPP3*, *EFEMP1*, *IGFBP4*, and *POSTN*) were selected with frequency at least 65% and the patient-level prediction remains stable across all subsets with size ≥40% (Supplementary Fig. 9d). Computationally, both peak memory usage and processing time displayed an almost linear increase with respect to different sizes of the input cells and samples (Supplementary Figs 7e and f, 8e and f, and 9e and f).

## Discussion

In this study, we present scPanel, a computational framework designed with three core functions: (i) identifying cell types responsive to perturbations; (ii) uncovering a minimal set of genes predictive of these perturbations; and (iii) enabling patient-level perturbation prediction in large-scale scRNA-seq datasets.

Recently, other methods, namely COMET and ActiveSVM, have been proposed to identify disease-specific gene sets and predict the patient’s disease state. However, these methods use all cells in the disease state as input, hence assuming that all diseased cells are homogeneous and the response to perturbation is also similar across all cells. This overlooks the fact that in some biological settings, only certain cell types may respond to perturbations. For example, in anti-PD1 treatment, only specific macrophage phenotypes (CCR2+ or MMP9+) are correlated with T cell expansion, an indicator of anti-PD1 treatment response, in pre-treatment tissues [49]. Thus, applying a uniform label to all cells, whether they are responding or non-responding counterparts, would dilute the biological signal originating from the responding macrophage subtypes. This makes it difficult for the model to converge and learn gene expression patterns specific to responding cell types. Recognizing the importance of accurately labelling responsive cells in diseased samples in the supervised learning process, scPanel emphasizes prioritizing the most responsive cell type for model training. Uniquely, scPanel addresses patient-level heterogeneity by training on one patient cohort and validating cell type responsiveness and gene importance scores in an unseen patient cohort. This approach ensured that the patient classification model by scPanel, trained with selected cell types and genes, is generalizable across different patients. To accommodate varying data complexities, five distinct algorithms were employed to develop the patient classification model, enhancing its robustness and applicability.

Transcriptomics data frequently exhibit multicollinearity, leading to gene modules comprising genes with similar expression patterns [50, 51]. Generally, using feature selection methods can mitigate this issue by removing redundant features [52]. In scPanel we proposed to implement SVM-RFE, a widely applied gene selection method in omics data [53–55], to reduce the redundancy caused by the multicollinearity in transcriptomics data. This results in finding a sparse gene panel to distinguish between two groups of patients. Empirically, we have benchmarked scPanel against COMET and ActiveSVM, as well as other feature selection algorithms, including filter methods, embedded methods and hybrid methods. The extensive benchmarking demonstrated that the wrapper-based SVM-RFE feature selection in the second step of scPanel provides a comparable or better performance in comparison with other methods tested (Fig. 7). The selection process is coupled with a cell-state classification task to make sure that the selected genes have enough predictive power.

Unlike other representation reduction methods such as Principal Component Analysis (PCA) or random composite measurements, scPanel returns interpretable genes instead of complex features such as linear combinations of genes [56]. If applied as a clinical tool, the predicted genes can be measured directly without developing complicated scoring metrics. Specifically, the identified sparse gene panel can be measured using targeted scRNA-seq capturing only a small number of genes, alleviating the problem of prohibitive sequencing costs. Alternatively, it is possible to design PCR-based test assays on sorted cell populations, i.e. focusing on a few genes from the most responsive cell type for clinical diagnosis. The measured expression levels of selected gene biomarkers are used to predict the patient status, which can then be compared with the predicted labels to validate the accuracy of the identified panel. Similar to the translation of the PAM50 signature to Prosigna™ [57], extensive tests in different patient cohorts will be necessary to confirm the assay's efficacy in clinical trials.

Single-cell data often reveal biological complexities across cell types as different cell populations may respond differently to perturbations. To handle this heterogeneity, scPanel identifies the most responsive cell type to use as input for biomarker selection and patient classification. This heuristic approach helps reducing the model complexity, and prevents the model from being affected by cell types that do not exhibit changes at the transcriptomic level, or by those for which the sample size is insufficient for accurate classification. When dealing with highly heterogeneous cell populations as in cancer, the cell-type selection step resolves this issue by identifying the cell type showing the largest difference between the two groups of patients while accounting for inter-patient heterogeneity within each group. As shown in the CRC case study, scPanel prioritized epithelial cells, which showed larger inter-group variation than intra-group heterogeneity. Subsequently, scPanel identified a sparse gene panel of nine genes that can accurately distinguish the patient subtype iCMS2 and iCMS3 with different prognosis.

A well-documented challenge with scRNA-seq data is the prevalence of batch effects [58, 59], where classifiers trained on one batch of patients significantly underperform on a separate, independently collected cohort due to technical disparities in sample collection, reagents, or sequencing platforms. This issue can be addressed by integrating the testing data with the training data to correct for batch effect. Ideally the batch effect correction method required by scPanel should fulfill three key criteria: (i) it should produce a batch-effect-corrected gene expression matrix (GEX) as input to the model; (ii) it must effectively preserve biological variance; and (iii) it should correct the testing data without altering the training data, thereby eliminating the need for model retraining. Among the various methods capable of producing corrected GEX as shown in recent benchmarking studies [60, 61], Seurat3 CCA and Seurat3 RPCA are the only two methods that can correct the testing data without modifying the training data. Seurat V3 CCA is particularly effective for removing batch effects where cell types are conserved across datasets [62]. However, despite its relatively good performance in preserving biological variance, Seurat3 CCA has been reported to suffer from overcorrection [63]. Thus, there is a need for the development of more advanced methods to achieve more accurate batch effect correction and improve patient classification. These methods can be integrated in the scPanel pipeline. Under the scenario of a good batch correction, we have shown that scPanel performs equally well across datasets from different studies (Fig. 5) or from studies with different conditions (Fig. 6). Notably, our analyses also reveal that the GAT classifier, a graph-based deep learning model, can predict well on different batches of data, despite being trained only on one batch. GAT, by design, leverages attention mechanisms to model relationships and dependencies between cells to output a more meaningful representation that can be generalized to different batches of data. The other four classification algorithms treat cells independently and find the optimal hyperplane that separates the different classes based on the original data, lacking the capacity to learn a more general representation as GAT.

Limitations. While scPanel provides a valuable framework for identifying sparse gene panels and classifying patients, it has several limitations. A clinically applicable gene panel ideally exhibits significant expression differences between groups, ensuring detectable signals by technologies like qRT-PCR. Thus, a future extension of scPanel would be incorporating the magnitude of gene expression differences into the gene selection process could enhance the panel’s discriminating power. To further improve the interpretability of scPanel in identifying biologically important genes, integrating a gene–gene correlation analysis function could help identify gene modules associated with each selected biomarker gene, which can then be annotated for their biological functions using public databases like MSigDB [64]. These functional gene modules can inform specific cellular processes involved in disease pathogenesis. Additionally, the high costs of scRNA-seq experiments still limit sample sizes in the range of tens to hundreds of samples, which might not comprehensively represent the entire population. Thus, the generalizability of gene panels derived from scPanel remains significantly limited by the size of input training data, which will eventually improve with the emergence of population-level single-cell studies encompassing more patients, and the development of multiplexing approaches [4]. Limitations in sample size also affect the generalizability. Here, the small number of patients (*n* = 6) used for external validation in the COVID-19 case study is not sufficient to guarantee 100% cross-dataset accuracy across datasets, and more external validations in larger datasets are required to demonstrate generalizability and quantify the accuracy of scPanel classification panel. Also, the scPanel-predicted gene panel may be affected by the presence of genes reflecting similar disease mechanisms; for example, as shown in the case of the COVID-19 gene panel that also predicts influenza cases. Thus, more comparative analyses using similar disease class single-cell data are necessary to further improve the specificity of the identified genes.

Besides scRNA-seq data, scPanel can be adapted to identify biomarkers from single cell proteomics data such as cytometry by time of flight [65]. Furthermore, we can harness the potential of deep learning, specifically through transfer learning, to enhance scPanel’s capabilities to predict on other data modalities. For example, we can use transfer learning to train on single cell transcriptomics data, and fine-tune on single cell proteomics data to enable prediction on patients when only single cell proteomics data are available. Beyond clinical application, scPanel can be used to test a priori research hypotheses, such as focusing on specific cell types or evaluating the importance of user-defined gene sets for patient classification.

Although scPanel currently focuses on binary classification, its future expansion to multi-class problems is feasible, given the availability of multi-class SVM-RFE for gene selection [66]. Specifically, for a k-class problem, k binary SVM classifiers are constructed to separate the class r from the other classes. For the r-th binary classification problem, SVM-RFE is carried out to identify a feature subset S_r_ for class r against all other classes. After k feature subsets are selected, the final selected subset for the whole multiclass problem is the combination of all the k subsets. For example, this would facilitate applications such as identifying biomarkers to predict tumor responses—complete response (CR), partial response (PR), stable disease (SD), and progressive disease (PD)—in accordance with Response Evaluation Criteria in Solid Tumors evaluation criteria. Other possible multi-class applications are to stratify patient disease subtypes or to predict treatment response to different therapies such as chemotherapy, targeted therapy, or combination therapy in cancer. scPanel can also be extended to use regression-based approaches to predict continuous outcomes, such as risk of heart failure or for identifying biomarkers that vary in association with aging. To handle such more complex multi-class problems, we believe that larger patient cohorts would be required to maintain the current accuracy and robustness observed in the simpler binary classification setting. Integrating additional information like cell type abundance and patient-level electronic health records could further refine the prediction accuracy. We anticipate that scPanel will be widely used, providing users a simple yet effective computational framework to prioritize sparse and interpretable gene panels from single-cell level data for potential clinical translation applications.

**Key Points**

- A machine learning–based framework, scPanel, for patient-level classification from scRNA-seq data.
- scPanel can identify responsive cell types and a sparse gene panel for patient classification.
- scPanel achieved high accuracy with small number of highly informative genes in scleroderma, CRC, and COVID-19 classification.
- Compared with existing approaches, the sparse biomarker genes panel selected by scPanel has higher predictive power.
- scPanel is available as an open-source Python package and implemented with parallel computing.

## Supplementary Material

Supplementary_Figures_bbae482

Supplementary_Methods_bbae482

Supplementary_Table_1_Summary_of_Datasets_bbae482

Supplementary_Table_2_Hyperparameters_used_in_classification_models_bbae482

## Data Availability

All scRNA-seq data used in the paper have been previously published. COVID-19 scRNA-seq data (wilk2020covid, su2020covid, lee2020cov_flu) are downloaded from COVID-19 Atlas (https://atlas.fredhutch.org/fredhutch/covid). Scleroderma scRNA-seq data (gur2022ssc) are downloaded from NCBI Gene Expression Omnibus (GEO) with accession number GEO: GSE195452. Colorectal cancer scRNA-seq data (joanito2022crc) are downloaded from Synapse with SynID:syn26844071 (https://www.synapse.org/#!Synapse:syn26844071/).

## Code Availability

Our method is integrated as an installable Python package called scPanel. The source code, installation instructions and user guidance are shown at https://github.com/carissaxie/scPanel.
