# Supplementary material for: scPanel: a tool for automatic identification of sparse gene panels for generalizable patient classification using scRNA-seq datasets
Source: Brief Bioinform. 2024 Sep 30;25(6):bbae482. doi: 10.1093/bib/bbae482 (PMC11442147; doi:10.1093/bib/bbae482)
Supplement: Supplementary_Methods_bbae482 [file supplementary_methods_bbae482.pdf]

# *Supplementary Methods for*

# scPanel: A tool for automatic identification of sparse gene panels for generalizable patient classification using scRNA-seq datasets

Yi Xie[1], Jianfei Yang[2], John F Ouyang[1*], Enrico Petretto[1*]

[1] Programme in Cardiovascular and Metabolic Disorders & Centre for Computational Biology, Duke-NUS Medical School, 8 College Road, 169857, Singapore

[2] the School of Mechanical and Aerospace Engineering and the School of Electrical and Electronic Engineering, Nanyang Technological University, Singapore 639798

## Design and implementation of scPanel

scPanel expects an input of normalized single cell gene expression matrix $D = \{x_{h,i}\}_{n \times K}$ where $x_{h,i}$ is the expression of gene $h$ in cell $i$. $n$ denotes the number of measured genes and $K$ denotes the number of cells. Each cell $i$ must have a sample label $Z_i$ indicating which sample this cell belongs to. Cell state labels $Y = \{y_1, y_2, \dots, y_i\}, y_i \in \{0,1\}$ are required and can be derived from the state of the sample the cells belong to (e.g. cells from COVID-19 patients are labeled as 1 and from healthy controls are labeled as 0). Importantly, it also expects that an initial cell type annotation of cells (denoted as $c$) is available. We denote the cell type labels as $c_i = t$, where $t \in \{1, \dots, T\}$ and $T$ is the total number of cell types in $D$.

Data is splitted into training set $D_{train}$, $Y_{train}$ and testing set $D_{test}$, $Y_{test}$ , with the division based on sample labels and stratification according to cell state labels. Z-score transformation is performed to standardize gene expression. To reduce the impact of technical outliers, the standardized values whose magnitude exceeds 10 are clipped. Gene expression mean and variance of the training data are used to standardize the testing data.

**Cell type selection**

For each cell type, samples with less than 20 cells (default) are removed. Cell types with less than 3 patients in at least one condition are removed. Responsive cells should become more separable from cells in control groups. scPanel qualifies responsiveness of cell types to perturbation by asking how readily the sample labels associated with each cell (for example, treatment versus control) can be predicted from the gene expression. In practice, we treat it as a classification problem. If cells in the treatment group are not responsive while being labeled as positive, the model will not converge during training. Thus, it will have low prediction performance during testing. We developed this functionality based on a random forest-based method, Augur.

Additionally, we conducted a comparison in terms of cell type responsive score (AUC) and computational cost (runtime) among random forest, XGBoost and CatBoost (Supplementary Figure 10, 11). Three algorithms demonstrated consistent AUC scores across all cell types. CatBoost is not preferred due to its substantially longer running time. Random Forest was selected as the classification algorithm included in this step considering it requires fewer hyperparameters to tune compared to XGBoost.

For each cell type at each iteration, we sampled (without replacement) 20 cells from each sample. 60% of samples are randomly splitted into the training set. Sample labels (for example, treatment versus control) are used as class labels to train a random forest classifier using scikit-learn with class_weight = 'balance' to avoid bias from different numbers of cells from different classes [1]. Classification performance of the model is evaluated on the remaining 40% samples and AUROC is calculated as the metrics to determine the responsiveness of cell types. This procedure is repeated 100 times by default for robust assessment. A Shapiro–Wilk test is carried out to test the normality of the distribution of AUROC values. If the p-value is larger than 0.05, the mean value is used to represent final AUROC for the cell type. Otherwise, the median value is used.

**Minimal gene panel selection**

SVM-RFE was introduced by Guyon et. al., for selecting important genes from gene expression data for cancer classification [2]. SVM-RFE, starting with all the genes, removes the gene that is least significant for classification recursively in a backward elimination manner. In each iteration, the ranking score of gene $s$ is computed from the coefficients of the weight vector $w$ of a linear SVM as follows:

$$s = w^2$$

$$w = \sum_i \alpha_i y_i x_i$$

Where $\boldsymbol{y_i} \in \boldsymbol{\ell}$ is the class label of the cell $x_i$ and the summation is taken over all the training samples. $\alpha_i$ are the Lagrange multipliers involved in maximizing the margin of separation of the classes. For computational efficiency, 3% of genes are removed at each iteration by default in scPanel. We allow users to adjust this value or remove one gene at a time by inputting different parameters.

The procedures of SVM-RFE are implemented as follows:

1. Start: ranked gene set $R = []$; selected gene subset $S = [1, \ldots, n]$;
2. Repeat until all genes are ranked:
   a. Given a training set $\{x_1, x_2, \ldots, x_i, \ldots, x_l\}$ and class labels $\{y_1, y_2, \ldots, y_i, \ldots, y_l\}$. Train a linear SVM with genes in set $S$ as input variables by minimizing:
   $$L = \frac{1}{2}\sum_{i=1}^{l}\sum_{j=1}^{l}\alpha_i\alpha_j y_i y_j (x_i \cdot x_j + \lambda\delta_{ij}) - \sum_{i=1}^{l}\alpha_i$$
   $$s.t. 0 \leq \alpha_i \leq C, \sum_{i=1}^{l} a_i y_i = 0$$
   $\delta_{ij}$ is the Kronecker symbol (if $i = j$ and 0 otherwise), and $\alpha = \{\alpha_1, \alpha_2, \ldots, \alpha_l\}$ are the parameters to be determined. $\lambda$ and $C$ are positive constants for regularization (soft margin parameters).
   b. Compute the weight vector;
   c. Compute the ranking scores for genes in set S: $s_i = (w_i)^2$;
   d. Find $0.03 \times n$ bottom-ranked genes $e = [1, \ldots, 0.03 \times n]$;
   e. Update: $R = [e, R], S = S - e$;
3. Output: Ranked feature list $R$.

To determine how many top-ranked genes in $R$ should be selected from the training data, we adopted SVM-RFE with a N-fold cross validation strategy (SVM-RFECV), where the training data are further partitioned into N equal-sized subsets (folds), N-1 folds are taken as the internal training set and the remaining one fold is taken as the internal validation set. At each fold, whenever the bottom-ranked gene was removed by SVM-RFE, the resulting gene subset was evaluated by summarizing the classification performance of the same SVM model on the internal validation set using AUPRC scores. The minimal number of genes is determined on which the SVM can get optimal AUPRC scores across N folds. The optimal AUPRC scores are determined in a data-driven way by applying perpendicular line method [3] on the parsimony plot where x-axis is the number of top-ranked genes retained in SVM-RFE in the internal training sets and y-axis represents the average classification performance of the same SVM model in the internal testing sets. scPanel automatically chooses the number of top-ranked genes $M$ with the longest perpendicular line to the line passing the two points of the highest and lowest ranks. Top $M$ genes from ranked feature list $R$ are taken as the minimal gene panel.

To avoid bias towards class or samples with more cells, we scale the parameter $C$ by multiplying with a cell factor $p_i$ in SVM loss function. So, a high value of $p_i$ means less regularization for the cell and a higher incentive for the SVM to classify it properly.

$$p_i = \frac{1}{u_i v_i}$$

Where $u_i$ is the class frequency of cell $k$, $v_i$ is the sample frequency of cell $i$

**Stable Gene Selection**

To enhance the robustness of gene selection, we incorporate a stable gene selection mode into scPanel. This approach involves the iterative downsampling of the training dataset without

replacement, creating multiple subsets. Each subset is then subjected to the scPanel's minimal gene panel selection algorithm. Genes frequently selected across these iterations are identified as the stable gene panel. We show this procedure in analyzing the severe COVID-19 dataset (wilk2020covid) [4], where we apply downsampling rates of 70%, 80%, and 90% to the training data, each iterating 20 times. Genes exhibiting a selection frequency exceeding 50% are designated as stable genes. We allow users to specify the downsampling rates, number of iterations, and selection frequency threshold in scPanel.

**Model training and Patient-level classification**

Given the selected most responsive cell type $c^*$ and minimal gene panel $R$. We subset the gene expression matrix $D$ and class label $Y$ into:

$$D_{R,c*} = D_{train}[R, c_i = c^*]$$

$$Y_{c*} = Y_{train}[c_i = c^*]$$

We use $D_{R,c*}$ and $Y_{c*}$ as inputs to train 5 different classifiers including:

Logistic Regression (LR): $f_{LR}$

Support Vector Machine (SVM): $f_{SVM}$

Random Forest (RF): $f_{RF}$

k-Nearest Neighbors (kNN): $f_{kNN}$

Graph Attention Network (GAT): $f_{GAT}$

For GAT, $D_{R,c*}$ was input to *compute_neighbors()* in scanpy python package to construct a cell-cell graph based on k-nearest neighbors (kNN). The resulting adjacency matrix, which indicates direct connections between nodes (cells) was used to construct the input graph to GAT. The gene expression values were used as node features in GAT.

During the testing phase, for each cell, we computed the final prediction outcome by taking the median of the prediction probabilities generated by the ensemble of the 5 classifiers.

- For LR it naturally outputs probabilities:

$$p_{LR} = f_{LR}$$

- For SVM, the prediction probability of each cell is estimated using Platt's method [5] as follows:

$$p_{i,svm} = \frac{1}{1 + exp(A \times f_{svm} + B)}$$

  where A and B are parameters (estimated by maximum likelihood) of sigmoid link function that converts the output $f_{SVM}$ from the SVM into a probability.

- For RF, the prediction probability of each cell is estimated from a collection of decision trees as follows:

$$p_{i,RF} = \frac{1}{N_{trees}} \sum_{b=1}^{N_{trees}} round(h(x_i|\theta_b))$$

  Where $h(x|\theta_b)$ denote decision tree, each parameterised by $\theta_b$ by training on the $b$th bootstrap data sample and a subset of features randomly sampled. The $round()$ function converts the probability value to the nearest integer to either 0 or 1. We estimate the probabilities by making a class prediction for each tree, and counting the fraction of trees that vote for a certain class.

- For kNN, it use the proportion of the k-nearest neighbors that belong to a certain class as the probability:

$$p_{i,kNN} = \frac{1}{k} \sum_{j \in Neighbors_k(i)} I(Y_{c*}[j] = 1)$$

where $Neighbors_k(i)$ denotes the set of indices of the k-nearest neighbors of the cell. The $I(.)$ function converts the true or false to either 1 or 0.

- For GAT, it outputs probabilities by considering the features of nodes (cells) and the graph structure. For a cell i, the probability of belonging to class 1 can be represented as:

$$p_{i,GAT} = \sigma\left(\sum_{j\in N(i)} \alpha_{ij} W D_{R,c*}\right)$$

where $N(i)$ is the set of neighbors of cell i, $\alpha_{ij}$ are the attention coefficients, $W$ is a weight matrix, and $\sigma$ is the sigmoid activation function.

After obtaining the prediction probabilities from each of the classifiers for a given cell, median of these probabilities is calculated as final prediction outcome for the given cell:

$$p_i = median\,(p_{i,LR}, p_{i,SVM}, p_{i,RF}, p_{i,kNN}, p_{i,GAT})$$

Subsequently, for each sample, we calculated the Area Under the Receiver Operating Characteristic (AUROC) curve. This was accomplished by first ranking the cells within the sample based on their median prediction probabilities. These ranks served as the x-axis, whereas the corresponding median probabilities were plotted on the y-axis. The area under this curve is computed as AUROC for each sample, serving as the measure of the patient-level classification performance. Sample with $AUROC \geq 0.5$ is predicted as 1, otherwise the sample is predicted as 0.

In order to evaluate the statistical significance of the sample-level predictions, we assessed whether the AUROC was significantly different from that of a random classifier, having an expected AUROC of 0.5. To achieve this, we employed a non-parametric bootstrap approach. As described in the last paragraph, we denoted the sample-level AUROC of the predictive model

computed on the testing data as $A_{sample}$. Under the null hypothesis that the model is no better than random guessing, we set $H_0: A = 0.5$. Subsequently, we generated $N$ bootstrap samples $\{D_1, D_2, \ldots, D_N\}$ from the original testing data. For each bootstrap sample $D_i$, the AUROC $A_i$ was computed, resulting in a distribution of AUROC values under the null hypothesis. The empirical p-value was calculated to quantify the significance of $A_{sample}$. It was defined as the proportion of bootstrap AUROC values that were as extreme as or more extreme than $A_{sample}$ represented as:

$$p = \frac{1}{N}\sum_{i=1}^{N} I(A_i \geq A_{sample})$$

where $I$ is an indicator function, yielding 1 if $A_i \geq A_{sample}$ and 0 otherwise. A small empirical p-value indicates that the observed AUROC is significantly different from what would be expected under the null hypothesis, hence suggesting that the predictive model performs better than random guessing.

**Data preprocessing**

Gene expression matrices were first columns normalized and log transformed with *NormalizeData()* in the Seurat R package. Genes that are expressed in at least one cell are retained in the data.

**Cross-batch prediction**

Testing data was integrated with training data using *Seurat V3 CCA* method [6]. *SelectIntegrationFeatures()* was used to find the top 2000 highly variable genes shared by both data as integration features. *FindIntegrationAnchors()* was used to find anchor features with parameter *n_PC = 20*. *IntegrateData()* was used for integration with parameters *n_PC = 20*, *k_weight = 100*.

**Benchmarking scPanel gene selection with other feature selection methods**

We compared the performance of scPanel gene selection procedures with state-of-the-art methods: (1) differentially expressed genes (DE genes) [6], (2) ActiveSVM (version 4.0.1) [7] and (3) COMET (version 0.1.13) [8]; Filter methods including (4) Pearson Correlation, (5) Mutual Information; Embedded methods including (6) Lasso, (7) Decision Tree; Hybrid method (8) naive-SVM.

For (1), *FindMarkers()* from the *Seurat* R package (version 4.4.0) was used to identify differentially expressed genes using a Wilcoxon Rank Sum test. Genes were ranked by absolute values of average log fold changes. For (2), Min-complexity mode was run with parameters *num_samples* = *100, init_samples= 200, balance=True* for COVID-19 dataset (wilk2020covid) and *num_samples = 200, init_samples = 400* for scleroderma dataset (gur2022ssc); Min-cell mode was run with parameters *num_samples = 100, init_samples = 200, balance=True* for COVID-19 dataset (wilk2020covid) and *num_samples = 200, init_samples = 400* for scleroderma dataset (gur2022ssc). (3) COMET was run with default parameters. For (4) – (8), *scikit-learn* (version 1.0.2) was used with default parameters.

In an attempt to provide a fair comparison, (i) we used the most responsive cell type identified by scPanel as the input data all the above mentioned methods for gene selection, ensuring that the same training data is provided to each method and (ii) the predicted gene panels by each method is then subjected to the same patient classification pipeline, implemented by scPanel, to report the patient AUROC as the final benchmarking metric.

For COMET, ActiveSVM, DE genes and filter methods, benchmarking is conducted by comparing the performance of patient classifiers trained with top 1, 2, …, K genes in each method’s ranking

list. We repeat this process 20 times, each time randomly selecting a subset of the training data that constitutes 50% of its original size. For embedded methods, which can automatically select gene panels, benchmarking involves comparing the sparsity of the gene panel and corresponding patient classification performance. This is also repeated 20 times by following the same sampling strategy.

Patient-level AUROC is used as the benchmarking metric. Specifically, the same training data was input to these methods to select genes. The patient-level classification model training and testing were uniformly conducted using the same parameters in scPanel. Patient-level AUROC is computed for each sample relative to its specific class in the testing data.

To complete our benchmark, computational cost (runtime) of scPanel is compared with SOTA gene selection methods, COMET and ActiveSVM. We increased the number of cells randomly sampled from the full dataset by 10% at each step and measured the running time of each method using a 16-core CPU.

## Empirical Power Analysis

The empirical power of scPanel was estimated with respect to the number of cells and the number of samples (scRNA-seq datasets), as follows. For cell-level power analysis, we downsampled the training set by cell to different proportions, each repeated for 20 times. The downsampling was done without replacement and stratified by sample and cell type. Sample-level power analysis was done similarly by downsampling the training set by sample to different proportions, each repeated for 20 times. The testing set remains the same. The downsampled training set is used to run scPanel for cell type selection, gene selection and patient-level classification.

**Assessment of the elapsed computational time and peak memory usage**

All power analysis tasks were performed on a research High Performance Computer (HPC) system with AMD EPYC 7763 64-Core Processors. 50 CPUs and 256 Gb total memory are allocated. The elapsed computational time was evaluated by the function '*time()*' from the *time* python module; timings for each method include all pre-processing steps. The usage of peak memory was monitored by the function '*memory_usage()*' from *memory_profiler* python module. Random seeds were fixed for all steps in the scPanel to ensure reproducibility.

# Reference

1. Pedregosa F, Varoquaux G, Gramfort A, et al. Scikit-learn: Machine Learning in Python. Journal of Machine Learning Research 2011; 12:2825–2830
2. Guyon I, Weston J, Barnhill S, et al. Gene Selection for Cancer Classification using Support Vector Machines. Machine Learning 2002; 46:389–422
3. Zhuang H, Wang H, Ji Z. findPC: An R package to automatically select the number of principal components in single-cell analysis. Bioinformatics 2022; 38:2949–2951
4. Wilk AJ, Rustagi A, Zhao NQ, et al. A single-cell atlas of the peripheral immune response in patients with severe COVID-19. Nat Med 2020; 26:1070–1076
5. Platt J. Probabilistic Outputs for Support vector Machines and Comparisons to Regularized Likelihood Methods. 1999;
6. Hao Y, Hao S, Andersen-Nissen E, et al. Integrated analysis of multimodal single-cell data. Cell 2021; 184:3573-3587.e29
7. Chen X, Chen S, Thomson M. Active feature selection discovers minimal gene-sets for classifying cell-types and disease states in single-cell mRNA-seq data. arXiv:2106.08317 [cs, q-bio] 2021;
8. Delaney C, Schnell A, Cammarata LV, et al. Combinatorial prediction of marker panels from single-cell transcriptomic data. Molecular Systems Biology 2019; 15:e9005
